# *trans*-Dichloridotetrakis­[1-(2-hydroxy­ethyl)-1*H*-tetrazole-κ*N* ^4^]cobalt(II)

**DOI:** 10.1107/S1600536809042263

**Published:** 2009-10-23

**Authors:** Alexander S. Lyakhov, Anastasiya P. Mosalkova, Mikhail M. Degtyarik, Ludmila S. Ivashkevich, Pavel N. Gaponik

**Affiliations:** a Research Institute for Physico-Chemical Problems, Belarusian State University, Leningradskaya Str. 14, Minsk 220030, Belarus

## Abstract

The title cobalt(II) complex, [CoCl_2_(C_3_H_6_N_4_O)_4_], was obtained from metallic cobalt by direct synthesis. There are two Co atoms in the asymmetric unit, each lying on an inversion centre and adopting a distorted octa­hedral coordination. Classical and non-classical hydrogen bonds are responsible for formation of a three-dimensional polymeric network in the crystal.

## Related literature

For a review of complexes of 1-substituted tetra­zoles, see: Gaponik *et al.* (2006 ▶). For the crystal structure of a related Co(II) complex, see: Shvedenkov *et al.* (2003 ▶). For a description of the Cambridge Structural Database, see: Allen (2002 ▶).
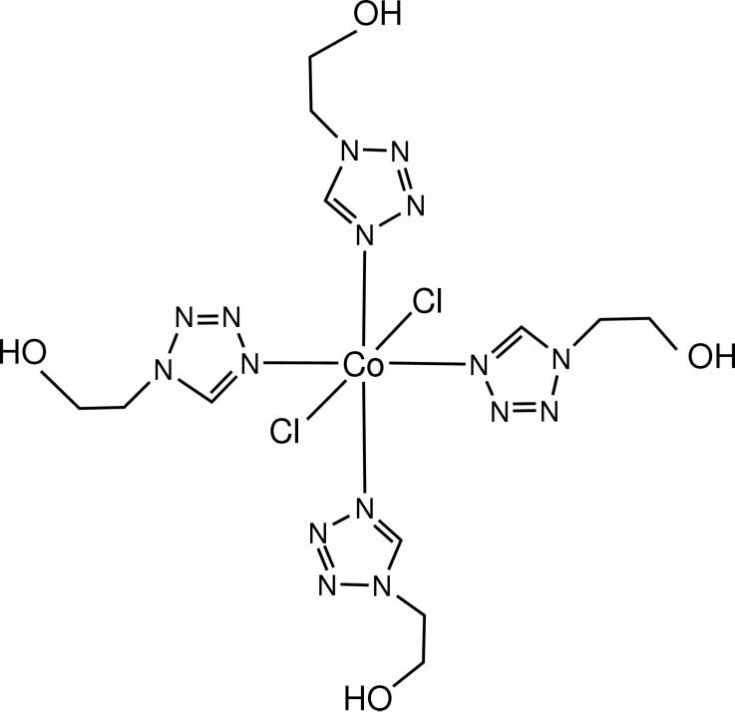


## Experimental

### 

#### Crystal data

[CoCl_2_(C_3_H_6_N_4_O)_4_]
*M* *_r_* = 586.30
Triclinic, 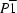

*a* = 6.8971 (19) Å
*b* = 9.4602 (17) Å
*c* = 19.761 (4) Å
α = 77.870 (14)°
β = 86.721 (19)°
γ = 69.481 (17)°
*V* = 1180.4 (5) Å^3^
*Z* = 2
Mo *K*α radiation
μ = 1.01 mm^−1^
*T* = 294 K
0.24 × 0.16 × 0.15 mm

#### Data collection

Nicolet R3m four-circle diffractometer
Absorption correction: ψ scan (North *et al.*, 1968 ▶) *T*_min_ = 0.794, *T*_max_ = 0.863
5904 measured reflections
5449 independent reflections
3747 reflections with *I* > 2σ(*I*)
*R*_int_ = 0.024
2 standard reflections every 100 reflections intensity decay: none

#### Refinement

*R*[*F* ^2^ > 2σ(*F* ^2^)] = 0.038
*wR*(*F* ^2^) = 0.097
*S* = 1.04
5449 reflections
323 parameters
H-atom parameters constrained
Δρ_max_ = 0.58 e Å^−3^
Δρ_min_ = −0.32 e Å^−3^

### 

Data collection: *R3m Software* (Nicolet, 1980 ▶); cell refinement: *R3m Software*; data reduction: *OMNIBUS* (Gałdecka, 2002 ▶); program(s) used to solve structure: *SIR2004* (Burla *et al.*, 2005 ▶); program(s) used to refine structure: *SHELXL97* (Sheldrick, 2008 ▶); molecular graphics: *PLATON* (Spek, 2009 ▶); software used to prepare material for publication: *SHELXL97* and *PLATON*.

## Supplementary Material

Crystal structure: contains datablocks global, I. DOI: 10.1107/S1600536809042263/dn2499sup1.cif

Structure factors: contains datablocks I. DOI: 10.1107/S1600536809042263/dn2499Isup2.hkl

Additional supplementary materials:  crystallographic information; 3D view; checkCIF report

## Figures and Tables

**Table 1:** Selected bond lengths (Å)

Co1—N24	2.144 (2)
Co1—N14	2.191 (2)
Co1—Cl1	2.4372 (10)
Co2—N44	2.165 (2)
Co2—N34	2.168 (2)
Co2—Cl2	2.4333 (10)

**Table 2:** Hydrogen-bond geometry (Å, °)

*D*—H⋯*A*	*D*—H	H⋯*A*	*D*⋯*A*	*D*—H⋯*A*
O1—H1⋯O3^i^	0.82	1.94	2.741 (3)	165
O2—H2⋯Cl1^ii^	0.82	2.32	3.104 (2)	160
O3—H3⋯Cl2^iii^	0.82	2.28	3.098 (3)	173
O4—H4⋯O2^iv^	0.82	1.94	2.757 (3)	175
C15—H15⋯Cl1^ii^	0.93	2.76	3.482 (3)	135
C25—H25⋯O1^v^	0.93	2.56	3.303 (4)	137
C35—H35⋯O4^vi^	0.93	2.38	3.156 (3)	141
C45—H45⋯Cl2^iii^	0.93	2.54	3.405 (3)	155

Symmetry codes: (i) 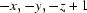; (ii) 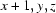; (iii) 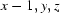; (iv) 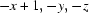; (v) 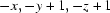; (vi) 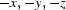.

**Fig. 1.**
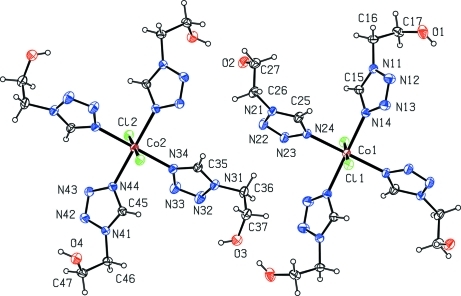
Two complex molecules in the crystal structure of (I), with atom numbering for the asymmetric unit. Displacement ellipsoids are drawn at the 30% probability level and H atoms are shown as spheres of arbitrary radii.

**Fig. 2.**
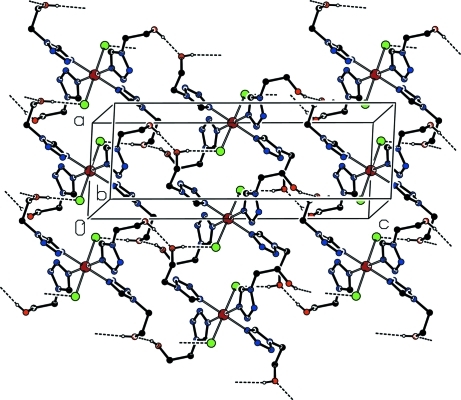
A layer in the structure of (I) parallel with the ac plane formed by classic hydrogen bonds O—H···O and O—H···Cl (dashed lines). Only H atoms, participating in classic hydrogen bonds, are shown.

**Fig. 3.**
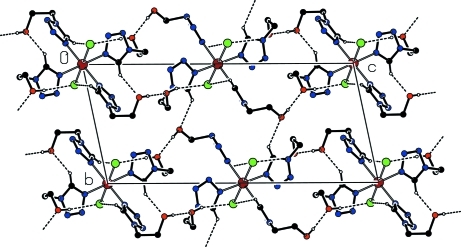
The crystal structure of (I) viewed along the a axis. Dashed lines show hydrogen bonds.

## supplementary crystallographic information

### Comment

Coordination compounds of 1-substituted tetrazoles have been the subject of many investigations (Gaponik *et al.*, 2006). Among reported metal(II) halide complexes containing 1-alkyltetrazoles overwhelming majority present copper(II) complexes Cu*L*_2_*X*_2_, where *L* = 1-alkyltetrazole, *X* = Cl or Br. Until now, only one cobalt(II) chloride complex with 1-allyltetrazole of composition Co*L*_2_Cl_2_ have been structurally characterized (Shvedenkov *et al.*, 2003). Here we present novel cobalt(II) chloride complex, namely Co*L*_4_Cl_2_ where *L* is 1-(2-hydroxyethyl)tetrazole, obtained by dissolving metallic cobalt in a methanol solution of 1-(2-hydroxyethyl)tetrazole in presence of hydrochloric acid in air. This is the first complex of such composition among metal(II) halide with 1-alkyltetrazoles obtained by now.

The title compound, (I), presents molecular complex, with two Co atoms in the asymmetric unit, both lying on inversion centres (Fig.1). Co atoms adopt rather distorted octahedral coordination composed of two Cl atoms in axial positions and four tetrazole ring N^4^ atoms in equatorial sites (Table 1). So, the complex molecules present *trans*-isomers.

The tetrazole ring geometry of ligand molecules is usual for 1-substituted tetrazoles (Cambridge Structural Database, Version 5.30 of November 2008; Allen, 2002).

The crystal packing of (I) is stabilized by a series of intermolecular hydrogen bonds (Table 2). Classic HB, O—H···O and O—H···Cl, link complex molecules to polymeric layers parallel to the *ac* plane (Fig. 2). Non-classic HB C^Tz^—H···Cl, formed by the tetrazole ring C—H groups, are additional within the layers. The above layers are connected *via* non-classic C^Tz^—H···O hydrogen bonds to give three-dimensional polymeric network (Fig. 3).

### Experimental

A mixture, containing cobalt powder (0.06 g, 0.001 mol), 1-(2-hydroxyethyl)tetrazole (0.47 g, 0.0041 mol), 5 ml of methanol and 0.2 ml of concentrated solution of HCl, was heated at 325 K with stirring on air until the metal was fully dissolved (6 h). Pink crystals of the title complex were grown by slow evaporation of the reaction mixture in air at room temperature during two weeks. The crystals were filtered off, washed with diethyl ether and dried in air (0.45 g, yield 85%; m.p. 383 K; decomp. 468 K). Calc. (%): Cu 10.1, Cl 12.1. Found (%): Cu 10.2, Cl 12.8. IR (cm^-1^): 3391 (*s*), 3091 (*s*), 3134 (*s*), 2896 (*m*), 2946 (*m*), 2975 (*s*), 1623 (*s*), 1497 (*s*), 1437 (*s*), 1380 (w), 1359 (*m*), 1275 (*m*), 1247 (w), 1171 (*s*), 1099 (*s*), 1062 (*s*), 998 (*s*), 259 (*s*), 200 (*m*), 176(*m*).

### Refinement

H atoms were placed in calculated positions and refined using riding model, with *U*_iso_(H)=1.2*U*_eq_(C) for the methylene and the tetrazole ring CH group, and *U*_iso_(H)=1.5*U*_eq_(O) for the hydroxyl groups.

### Crystal data

**Table:**

[CoCl_2_(C_3_H_6_N_4_O)_4_]	*Z* = 2
*M**_r_* = 586.30	*F*(000) = 602
Triclinic, *P*1	*D*_x_ = 1.650 Mg m^−^^3^
Hall symbol: -P 1	Melting point: 383 K
*a* = 6.8971 (19) Å	Mo *K*α radiation, λ = 0.71073 Å
*b* = 9.4602 (17) Å	Cell parameters from 25 reflections
*c* = 19.761 (4) Å	θ = 13.4–19.5°
α = 77.870 (14)°	µ = 1.01 mm^−^^1^
β = 86.721 (19)°	*T* = 294 K
γ = 69.481 (17)°	Prism, pink
*V* = 1180.4 (5) Å^3^	0.24 × 0.16 × 0.15 mm

### Data collection

**Table:**

Nicolet R3m four-circle diffractometer	3747 reflections with *I* > 2σ(*I*)
Radiation source: fine-focus sealed tube	*R*_int_ = 0.024
graphite	θ_max_ = 27.6°, θ_min_ = 1.1°
ω/2θ scans	*h* = 0→8
Absorption correction: ψ scan (North *et al.*, 1968)	*k* = −11→12
*T*_min_ = 0.794, *T*_max_ = 0.863	*l* = −25→25
5904 measured reflections	2 standard reflections every 100 reflections
5449 independent reflections	intensity decay: none

### Refinement

**Table:**

Refinement on *F*^2^	Primary atom site location: structure-invariant direct methods
Least-squares matrix: full	Secondary atom site location: difference Fourier map
*R*[*F*^2^ > 2σ(*F*^2^)] = 0.038	Hydrogen site location: inferred from neighbouring sites
*wR*(*F*^2^) = 0.097	H-atom parameters constrained
*S* = 1.04	*w* = 1/[σ^2^(*F*_o_^2^) + (0.031*P*)^2^ + 0.9939*P*] where *P* = (*F*_o_^2^ + 2*F*_c_^2^)/3
5449 reflections	(Δ/σ)_max_ < 0.001
323 parameters	Δρ_max_ = 0.58 e Å^−^^3^
0 restraints	Δρ_min_ = −0.32 e Å^−^^3^

### Special details

**Table:**

Geometry. All e.s.d.'s (except the e.s.d. in the dihedral angle between two l.s. planes) are estimated using the full covariance matrix. The cell e.s.d.'s are taken into account individually in the estimation of e.s.d.'s in distances, angles and torsion angles; correlations between e.s.d.'s in cell parameters are only used when they are defined by crystal symmetry. An approximate (isotropic) treatment of cell e.s.d.'s is used for estimating e.s.d.'s involving l.s. planes.
Refinement. Refinement of *F*^2^ against ALL reflections. The weighted *R*-factor *wR* and goodness of fit *S* are based on *F*^2^, conventional *R*-factors *R* are based on *F*, with *F* set to zero for negative *F*^2^. The threshold expression of *F*^2^ > σ(*F*^2^) is used only for calculating *R*-factors(gt) *etc*. and is not relevant to the choice of reflections for refinement. *R*-factors based on *F*^2^ are statistically about twice as large as those based on *F*, and *R*- factors based on ALL data will be even larger.

### Fractional atomic coordinates and isotropic or equivalent isotropic displacement parameters (Å^2^)

**Table:**

	*x*	*y*	*z*	*U*_iso_*/*U*_eq_	
Co1	0.0000	0.0000	0.5000	0.02622 (12)	
Cl1	−0.33214 (10)	0.17425 (8)	0.44772 (3)	0.03638 (16)	
N11	0.1924 (3)	0.3322 (2)	0.56697 (11)	0.0329 (5)	
N12	−0.0116 (4)	0.3971 (3)	0.57773 (15)	0.0471 (6)	
N13	−0.1006 (4)	0.3080 (3)	0.56183 (14)	0.0450 (6)	
N14	0.0441 (3)	0.1851 (2)	0.54069 (11)	0.0326 (5)	
C15	0.2219 (4)	0.2040 (3)	0.54426 (14)	0.0342 (6)	
H15	0.3491	0.1380	0.5327	0.041*	
C16	0.3466 (4)	0.3983 (3)	0.58033 (15)	0.0389 (6)	
H16A	0.2933	0.5089	0.5631	0.047*	
H16B	0.4718	0.3556	0.5555	0.047*	
C17	0.3979 (5)	0.3655 (3)	0.65626 (16)	0.0445 (7)	
H17A	0.4320	0.2561	0.6750	0.053*	
H17B	0.5183	0.3923	0.6624	0.053*	
O1	0.2309 (4)	0.4496 (2)	0.69294 (12)	0.0564 (6)	
H1	0.1933	0.3896	0.7222	0.085*	
N21	0.2420 (3)	0.1961 (3)	0.31580 (11)	0.0320 (5)	
N22	0.3564 (4)	0.0462 (3)	0.32073 (13)	0.0455 (6)	
N23	0.2998 (4)	−0.0278 (3)	0.37622 (13)	0.0417 (6)	
N24	0.1491 (3)	0.0717 (2)	0.40773 (11)	0.0312 (5)	
C25	0.1170 (4)	0.2095 (3)	0.36908 (14)	0.0369 (6)	
H25	0.0219	0.3013	0.3778	0.044*	
C26	0.2763 (5)	0.3161 (3)	0.26103 (14)	0.0417 (7)	
H26A	0.1492	0.4045	0.2521	0.050*	
H26B	0.3132	0.2770	0.2187	0.050*	
C27	0.4452 (5)	0.3655 (3)	0.28125 (16)	0.0458 (7)	
H27A	0.4527	0.4530	0.2468	0.055*	
H27B	0.4124	0.3981	0.3252	0.055*	
O2	0.6405 (3)	0.2449 (3)	0.28720 (11)	0.0480 (5)	
H2	0.6670	0.2039	0.3281	0.072*	
Co2	0.5000	0.0000	0.0000	0.02837 (12)	
Cl2	0.84297 (10)	−0.16973 (8)	0.04419 (4)	0.03764 (16)	
N31	0.1868 (4)	−0.1224 (3)	0.18430 (12)	0.0368 (5)	
N32	0.3021 (4)	−0.2628 (3)	0.17307 (14)	0.0507 (7)	
N33	0.4073 (4)	−0.2413 (3)	0.11812 (14)	0.0472 (7)	
N34	0.3617 (3)	−0.0878 (2)	0.09318 (11)	0.0334 (5)	
C35	0.2248 (4)	−0.0173 (3)	0.13501 (14)	0.0337 (6)	
H35	0.1646	0.0889	0.1308	0.040*	
C36	0.0403 (5)	−0.1038 (4)	0.24140 (15)	0.0464 (7)	
H36A	0.0348	−0.0141	0.2589	0.056*	
H36B	0.0895	−0.1933	0.2787	0.056*	
C37	−0.1743 (5)	−0.0850 (4)	0.21924 (17)	0.0511 (8)	
H37A	−0.2701	−0.0556	0.2559	0.061*	
H37B	−0.2174	−0.0028	0.1786	0.061*	
O3	−0.1834 (5)	−0.2206 (3)	0.20404 (12)	0.0682 (7)	
H3	−0.1787	−0.2147	0.1620	0.102*	
N41	0.3421 (3)	−0.3452 (2)	−0.06627 (11)	0.0318 (5)	
N42	0.5298 (4)	−0.3745 (3)	−0.09471 (13)	0.0443 (6)	
N43	0.6031 (4)	−0.2741 (3)	−0.08241 (13)	0.0406 (6)	
N44	0.4653 (3)	−0.1786 (2)	−0.04598 (11)	0.0326 (5)	
C45	0.3046 (4)	−0.2248 (3)	−0.03711 (14)	0.0346 (6)	
H45	0.1844	−0.1801	−0.0141	0.041*	
C46	0.2061 (5)	−0.4333 (3)	−0.07074 (14)	0.0377 (6)	
H46A	0.0768	−0.3897	−0.0481	0.045*	
H46B	0.2717	−0.5391	−0.0466	0.045*	
C47	0.1617 (5)	−0.4311 (3)	−0.14470 (14)	0.0386 (6)	
H47A	0.2876	−0.4887	−0.1655	0.046*	
H47B	0.0607	−0.4807	−0.1459	0.046*	
O4	0.0844 (3)	−0.2775 (2)	−0.18342 (11)	0.0432 (5)	
H4	0.1605	−0.2673	−0.2161	0.065*	

### Atomic displacement parameters (Å^2^)

**Table:**

	*U*^11^	*U*^22^	*U*^33^	*U*^12^	*U*^13^	*U*^23^
Co1	0.0253 (2)	0.0280 (2)	0.0256 (2)	−0.0109 (2)	0.00245 (19)	−0.00379 (19)
Cl1	0.0302 (3)	0.0385 (4)	0.0346 (3)	−0.0074 (3)	−0.0020 (3)	−0.0023 (3)
N11	0.0332 (12)	0.0315 (11)	0.0356 (12)	−0.0121 (10)	0.0050 (9)	−0.0098 (9)
N12	0.0347 (13)	0.0423 (14)	0.0682 (18)	−0.0114 (11)	0.0058 (12)	−0.0246 (13)
N13	0.0314 (13)	0.0444 (14)	0.0629 (17)	−0.0121 (11)	0.0078 (12)	−0.0223 (13)
N14	0.0310 (12)	0.0320 (12)	0.0363 (12)	−0.0118 (9)	0.0034 (9)	−0.0093 (9)
C15	0.0302 (14)	0.0343 (14)	0.0410 (15)	−0.0115 (11)	0.0062 (11)	−0.0147 (12)
C16	0.0406 (16)	0.0359 (15)	0.0484 (17)	−0.0213 (13)	0.0058 (13)	−0.0136 (13)
C17	0.0465 (18)	0.0400 (16)	0.0512 (18)	−0.0150 (14)	−0.0017 (14)	−0.0178 (14)
O1	0.0714 (16)	0.0409 (12)	0.0480 (13)	−0.0071 (11)	0.0100 (11)	−0.0142 (10)
N21	0.0338 (12)	0.0337 (12)	0.0284 (11)	−0.0146 (10)	0.0032 (9)	−0.0016 (9)
N22	0.0558 (16)	0.0366 (13)	0.0438 (14)	−0.0174 (12)	0.0207 (12)	−0.0105 (11)
N23	0.0475 (14)	0.0316 (12)	0.0446 (14)	−0.0139 (11)	0.0174 (11)	−0.0092 (10)
N24	0.0310 (11)	0.0307 (11)	0.0305 (11)	−0.0102 (9)	0.0078 (9)	−0.0057 (9)
C25	0.0343 (14)	0.0309 (14)	0.0384 (15)	−0.0074 (11)	0.0066 (12)	−0.0005 (11)
C26	0.0446 (17)	0.0445 (16)	0.0321 (14)	−0.0195 (13)	0.0014 (12)	0.0074 (12)
C27	0.057 (2)	0.0396 (16)	0.0419 (17)	−0.0244 (15)	0.0012 (14)	0.0018 (13)
O2	0.0466 (12)	0.0555 (13)	0.0414 (12)	−0.0228 (11)	0.0020 (10)	0.0001 (10)
Co2	0.0272 (3)	0.0275 (3)	0.0326 (3)	−0.0113 (2)	0.0062 (2)	−0.0092 (2)
Cl2	0.0288 (3)	0.0396 (4)	0.0415 (4)	−0.0086 (3)	0.0040 (3)	−0.0082 (3)
N31	0.0374 (13)	0.0353 (12)	0.0340 (12)	−0.0121 (10)	0.0061 (10)	−0.0018 (10)
N32	0.0539 (16)	0.0317 (13)	0.0546 (16)	−0.0084 (12)	0.0135 (13)	0.0024 (11)
N33	0.0461 (15)	0.0292 (12)	0.0563 (16)	−0.0068 (11)	0.0165 (12)	−0.0026 (11)
N34	0.0327 (12)	0.0285 (11)	0.0373 (12)	−0.0105 (9)	0.0061 (9)	−0.0044 (9)
C35	0.0362 (14)	0.0294 (13)	0.0345 (14)	−0.0120 (11)	0.0069 (11)	−0.0054 (11)
C36	0.0562 (19)	0.0509 (18)	0.0338 (15)	−0.0231 (15)	0.0149 (14)	−0.0082 (13)
C37	0.0527 (19)	0.057 (2)	0.0509 (19)	−0.0274 (16)	0.0196 (15)	−0.0163 (16)
O3	0.106 (2)	0.0774 (17)	0.0483 (14)	−0.0632 (17)	0.0223 (15)	−0.0207 (13)
N41	0.0370 (12)	0.0334 (12)	0.0285 (11)	−0.0157 (10)	0.0021 (9)	−0.0080 (9)
N42	0.0465 (15)	0.0453 (14)	0.0502 (15)	−0.0205 (12)	0.0122 (12)	−0.0242 (12)
N43	0.0400 (13)	0.0427 (13)	0.0458 (14)	−0.0188 (11)	0.0124 (11)	−0.0189 (11)
N44	0.0311 (12)	0.0354 (12)	0.0348 (12)	−0.0129 (10)	0.0043 (9)	−0.0130 (10)
C45	0.0324 (14)	0.0365 (14)	0.0391 (15)	−0.0133 (12)	0.0056 (11)	−0.0162 (12)
C46	0.0491 (17)	0.0352 (14)	0.0354 (14)	−0.0233 (13)	0.0021 (12)	−0.0066 (11)
C47	0.0484 (17)	0.0319 (14)	0.0384 (15)	−0.0169 (13)	−0.0031 (13)	−0.0070 (12)
O4	0.0442 (12)	0.0343 (10)	0.0431 (12)	−0.0087 (9)	0.0006 (9)	0.0006 (9)

### Geometric parameters (Å, °)

**Table:**

Co1—N24	2.144 (2)	Co2—N44^ii^	2.165 (2)
Co1—N24^i^	2.144 (2)	Co2—N44	2.165 (2)
Co1—N14^i^	2.191 (2)	Co2—N34^ii^	2.168 (2)
Co1—N14	2.191 (2)	Co2—N34	2.168 (2)
Co1—Cl1^i^	2.4372 (10)	Co2—Cl2^ii^	2.4333 (10)
Co1—Cl1	2.4372 (10)	Co2—Cl2	2.4333 (10)
N11—C15	1.326 (3)	N31—C35	1.325 (3)
N11—N12	1.348 (3)	N31—N32	1.345 (3)
N11—C16	1.469 (3)	N31—C36	1.467 (3)
N12—N13	1.297 (3)	N32—N33	1.292 (3)
N13—N14	1.366 (3)	N33—N34	1.359 (3)
N14—C15	1.308 (3)	N34—C35	1.312 (3)
C15—H15	0.9300	C35—H35	0.9300
C16—C17	1.504 (4)	C36—C37	1.507 (4)
C16—H16A	0.9700	C36—H36A	0.9700
C16—H16B	0.9700	C36—H36B	0.9700
C17—O1	1.410 (3)	C37—O3	1.400 (4)
C17—H17A	0.9700	C37—H37A	0.9700
C17—H17B	0.9700	C37—H37B	0.9700
O1—H1	0.8200	O3—H3	0.8200
N21—C25	1.320 (3)	N41—C45	1.322 (3)
N21—N22	1.346 (3)	N41—N42	1.342 (3)
N21—C26	1.469 (3)	N41—C46	1.474 (3)
N22—N23	1.290 (3)	N42—N43	1.290 (3)
N23—N24	1.356 (3)	N43—N44	1.353 (3)
N24—C25	1.316 (3)	N44—C45	1.318 (3)
C25—H25	0.9300	C45—H45	0.9300
C26—C27	1.500 (4)	C46—C47	1.505 (4)
C26—H26A	0.9700	C46—H46A	0.9700
C26—H26B	0.9700	C46—H46B	0.9700
C27—O2	1.419 (4)	C47—O4	1.421 (3)
C27—H27A	0.9700	C47—H47A	0.9700
C27—H27B	0.9700	C47—H47B	0.9700
O2—H2	0.8200	O4—H4	0.8200
			
N24—Co1—N24^i^	180.00 (10)	N44^ii^—Co2—N44	180.000 (1)
N24—Co1—N14^i^	92.41 (8)	N44^ii^—Co2—N34^ii^	88.52 (8)
N24^i^—Co1—N14^i^	87.59 (8)	N44—Co2—N34^ii^	91.48 (8)
N24—Co1—N14	87.59 (8)	N44^ii^—Co2—N34	91.48 (8)
N24^i^—Co1—N14	92.41 (8)	N44—Co2—N34	88.52 (8)
N14^i^—Co1—N14	180.0	N34^ii^—Co2—N34	180.00 (8)
N24—Co1—Cl1^i^	91.04 (6)	N44^ii^—Co2—Cl2^ii^	90.56 (6)
N24^i^—Co1—Cl1^i^	88.96 (6)	N44—Co2—Cl2^ii^	89.45 (6)
N14^i^—Co1—Cl1^i^	91.03 (6)	N34^ii^—Co2—Cl2^ii^	90.42 (6)
N14—Co1—Cl1^i^	88.97 (6)	N34—Co2—Cl2^ii^	89.58 (6)
N24—Co1—Cl1	88.96 (6)	N44^ii^—Co2—Cl2	89.45 (6)
N24^i^—Co1—Cl1	91.04 (6)	N44—Co2—Cl2	90.55 (6)
N14^i^—Co1—Cl1	88.97 (6)	N34^ii^—Co2—Cl2	89.58 (6)
N14—Co1—Cl1	91.03 (6)	N34—Co2—Cl2	90.42 (6)
Cl1^i^—Co1—Cl1	180.00 (2)	Cl2^ii^—Co2—Cl2	180.0
C15—N11—N12	108.2 (2)	C35—N31—N32	108.4 (2)
C15—N11—C16	128.7 (2)	C35—N31—C36	130.1 (2)
N12—N11—C16	123.1 (2)	N32—N31—C36	121.4 (2)
N13—N12—N11	106.7 (2)	N33—N32—N31	106.8 (2)
N12—N13—N14	109.9 (2)	N32—N33—N34	109.9 (2)
C15—N14—N13	105.9 (2)	C35—N34—N33	106.3 (2)
C15—N14—Co1	124.65 (18)	C35—N34—Co2	131.63 (18)
N13—N14—Co1	129.21 (17)	N33—N34—Co2	122.09 (17)
N14—C15—N11	109.3 (2)	N34—C35—N31	108.7 (2)
N14—C15—H15	125.4	N34—C35—H35	125.7
N11—C15—H15	125.4	N31—C35—H35	125.7
N11—C16—C17	111.7 (2)	N31—C36—C37	112.1 (3)
N11—C16—H16A	109.3	N31—C36—H36A	109.2
C17—C16—H16A	109.3	C37—C36—H36A	109.2
N11—C16—H16B	109.3	N31—C36—H36B	109.2
C17—C16—H16B	109.3	C37—C36—H36B	109.2
H16A—C16—H16B	107.9	H36A—C36—H36B	107.9
O1—C17—C16	111.5 (3)	O3—C37—C36	112.0 (3)
O1—C17—H17A	109.3	O3—C37—H37A	109.2
C16—C17—H17A	109.3	C36—C37—H37A	109.2
O1—C17—H17B	109.3	O3—C37—H37B	109.2
C16—C17—H17B	109.3	C36—C37—H37B	109.2
H17A—C17—H17B	108.0	H37A—C37—H37B	107.9
C17—O1—H1	109.5	C37—O3—H3	109.5
C25—N21—N22	108.5 (2)	C45—N41—N42	108.1 (2)
C25—N21—C26	129.8 (2)	C45—N41—C46	128.3 (2)
N22—N21—C26	121.5 (2)	N42—N41—C46	123.6 (2)
N23—N22—N21	106.7 (2)	N43—N42—N41	107.2 (2)
N22—N23—N24	110.2 (2)	N42—N43—N44	109.9 (2)
C25—N24—N23	105.9 (2)	C45—N44—N43	106.0 (2)
C25—N24—Co1	130.62 (18)	C45—N44—Co2	124.45 (17)
N23—N24—Co1	123.40 (16)	N43—N44—Co2	129.29 (17)
N24—C25—N21	108.8 (2)	N44—C45—N41	108.9 (2)
N24—C25—H25	125.6	N44—C45—H45	125.6
N21—C25—H25	125.6	N41—C45—H45	125.6
N21—C26—C27	111.3 (2)	N41—C46—C47	111.7 (2)
N21—C26—H26A	109.4	N41—C46—H46A	109.3
C27—C26—H26A	109.4	C47—C46—H46A	109.3
N21—C26—H26B	109.4	N41—C46—H46B	109.3
C27—C26—H26B	109.4	C47—C46—H46B	109.3
H26A—C26—H26B	108.0	H46A—C46—H46B	108.0
O2—C27—C26	111.9 (3)	O4—C47—C46	110.8 (2)
O2—C27—H27A	109.2	O4—C47—H47A	109.5
C26—C27—H27A	109.2	C46—C47—H47A	109.5
O2—C27—H27B	109.2	O4—C47—H47B	109.5
C26—C27—H27B	109.2	C46—C47—H47B	109.5
H27A—C27—H27B	107.9	H47A—C47—H47B	108.1
C27—O2—H2	109.5	C47—O4—H4	109.5
			
C15—N11—N12—N13	0.2 (3)	C35—N31—N32—N33	0.2 (3)
C16—N11—N12—N13	−179.0 (2)	C36—N31—N32—N33	177.3 (3)
N11—N12—N13—N14	0.0 (3)	N31—N32—N33—N34	−0.1 (4)
N12—N13—N14—C15	−0.2 (3)	N32—N33—N34—C35	−0.1 (3)
N12—N13—N14—Co1	−175.40 (19)	N32—N33—N34—Co2	−178.8 (2)
N24—Co1—N14—C15	−53.8 (2)	N44^ii^—Co2—N34—C35	41.8 (3)
N24^i^—Co1—N14—C15	126.2 (2)	N44—Co2—N34—C35	−138.2 (3)
Cl1^i^—Co1—N14—C15	37.3 (2)	Cl2^ii^—Co2—N34—C35	−48.7 (3)
Cl1—Co1—N14—C15	−142.7 (2)	Cl2—Co2—N34—C35	131.3 (3)
N24—Co1—N14—N13	120.6 (2)	N44^ii^—Co2—N34—N33	−139.8 (2)
N24^i^—Co1—N14—N13	−59.4 (2)	N44—Co2—N34—N33	40.2 (2)
Cl1^i^—Co1—N14—N13	−148.4 (2)	Cl2^ii^—Co2—N34—N33	129.6 (2)
Cl1—Co1—N14—N13	31.6 (2)	Cl2—Co2—N34—N33	−50.4 (2)
N13—N14—C15—N11	0.4 (3)	N33—N34—C35—N31	0.2 (3)
Co1—N14—C15—N11	175.83 (16)	Co2—N34—C35—N31	178.73 (19)
N12—N11—C15—N14	−0.4 (3)	N32—N31—C35—N34	−0.2 (3)
C16—N11—C15—N14	178.8 (2)	C36—N31—C35—N34	−177.0 (3)
C15—N11—C16—C17	−101.2 (3)	C35—N31—C36—C37	84.0 (4)
N12—N11—C16—C17	77.8 (3)	N32—N31—C36—C37	−92.4 (3)
N11—C16—C17—O1	−70.2 (3)	N31—C36—C37—O3	68.6 (3)
C25—N21—N22—N23	−0.1 (3)	C45—N41—N42—N43	0.3 (3)
C26—N21—N22—N23	175.4 (3)	C46—N41—N42—N43	178.5 (2)
N21—N22—N23—N24	0.2 (3)	N41—N42—N43—N44	0.0 (3)
N22—N23—N24—C25	−0.2 (3)	N42—N43—N44—C45	−0.4 (3)
N22—N23—N24—Co1	177.84 (19)	N42—N43—N44—Co2	173.47 (19)
N14^i^—Co1—N24—C25	131.3 (3)	N34^ii^—Co2—N44—C45	−141.5 (2)
N14—Co1—N24—C25	−48.7 (3)	N34—Co2—N44—C45	38.5 (2)
Cl1^i^—Co1—N24—C25	−137.6 (3)	Cl2^ii^—Co2—N44—C45	−51.1 (2)
Cl1—Co1—N24—C25	42.4 (3)	Cl2—Co2—N44—C45	128.9 (2)
N14^i^—Co1—N24—N23	−46.2 (2)	N34^ii^—Co2—N44—N43	45.6 (2)
N14—Co1—N24—N23	133.8 (2)	N34—Co2—N44—N43	−134.4 (2)
Cl1^i^—Co1—N24—N23	44.8 (2)	Cl2^ii^—Co2—N44—N43	136.0 (2)
Cl1—Co1—N24—N23	−135.2 (2)	Cl2—Co2—N44—N43	−44.0 (2)
N23—N24—C25—N21	0.2 (3)	N43—N44—C45—N41	0.6 (3)
Co1—N24—C25—N21	−177.68 (18)	Co2—N44—C45—N41	−173.65 (17)
N22—N21—C25—N24	−0.1 (3)	N42—N41—C45—N44	−0.6 (3)
C26—N21—C25—N24	−175.1 (3)	C46—N41—C45—N44	−178.6 (2)
C25—N21—C26—C27	89.6 (4)	C45—N41—C46—C47	120.9 (3)
N22—N21—C26—C27	−84.9 (3)	N42—N41—C46—C47	−56.9 (3)
N21—C26—C27—O2	65.8 (3)	N41—C46—C47—O4	−53.2 (3)

Symmetry codes: (i) −*x*, −*y*, −*z*+1; (ii) −*x*+1, −*y*, −*z*.

### Hydrogen-bond geometry (Å, °)

**Table:**

*D*—H···*A*	*D*—H	H···*A*	*D*···*A*	*D*—H···*A*
O1—H1···O3^i^	0.82	1.94	2.741 (3)	165
O2—H2···Cl1^iii^	0.82	2.32	3.104 (2)	160
O3—H3···Cl2^iv^	0.82	2.28	3.098 (3)	173
O4—H4···O2^ii^	0.82	1.94	2.757 (3)	175
C15—H15···Cl1^iii^	0.93	2.76	3.482 (3)	135
C25—H25···O1^v^	0.93	2.56	3.303 (4)	137
C35—H35···O4^vi^	0.93	2.38	3.156 (3)	141
C45—H45···Cl2^iv^	0.93	2.54	3.405 (3)	155

Symmetry codes: (i) −*x*, −*y*, −*z*+1; (iii) *x*+1, *y*, *z*; (iv) *x*−1, *y*, *z*; (ii) −*x*+1, −*y*, −*z*; (v) −*x*, −*y*+1, −*z*+1; (vi) −*x*, −*y*, −*z*.
